# 28 NICUs participating in a quality improvement collaborative targeting early-onset sepsis antibiotic use

**DOI:** 10.1038/s41372-024-01885-8

**Published:** 2024-02-20

**Authors:** Kurlen S. E. Payton, Mihoko V. Bennett, Joseph Schulman, William E. Benitz, Lisa Stellwagen, Gary L. Darmstadt, Jenny Quinn, Alexandria I. Kristensen-Cabrera, Courtney C. Breault, Michael Bolaris, Linda Lefrak, Jeff Merrill, Paul J. Sharek

**Affiliations:** 1 Department of Pediatrics, Division of Neonatology, Cedars-Sinai Medical Center, Los Angeles, CA USA; 2 California Perinatal Quality Care Collaborative (CPQCC), Stanford, CA USA; 3 Department of Pediatrics, Division of Neonatal and Developmental Medicine, Stanford University School of Medicine, Stanford, CA USA; 4 CA Department of Health Care Services, California Children’s Services, Sacramento, CA USA; 5 Department of Pediatrics, Division of Academic General Pediatrics, University of California San Diego School of Medicine, San Diego, CA USA; 6 Prematurity Research Center, Department of Pediatrics, Division of Neonatal and Developmental Medicine, Stanford University School of Medicine, Stanford, CA USA; 7 Department of Pediatrics, Division of Infectious Disease, Harbor-University of California Los Angeles Medical Center, Los Angeles, CA USA; 8 California Department of Public Health, Center for Health Care Quality, Health Care Associated Infections Program, Richmond, CA USA; 9 Sutter Health Summit Medical Center, Oakland, CA USA; 10 Department of Pediatrics, Division of Hospitalist Medicine, Stanford University School of Medicine, Stanford, CA USA; 11 Department of Pediatrics, Division of Hospitalist Medicine, University of Washington School of Medicine, Seattle, WA USA; 12 Center for Quality and Patient Safety, Seattle Children’s Hospital, Seattle, WA USA

**Keywords:** Paediatrics, Antimicrobial therapy

## Abstract

**Objective:**

There is widespread overuse of antibiotics in neonatal intensive care units (NICUs). The objective of this study was to safely reduce antibiotic use in participating NICUs by targeting early-onset sepsis (EOS) management.

**Study design:**

Twenty-eight NICUs participated in this statewide multicenter antibiotic stewardship quality improvement collaborative. The primary aim was to reduce the total monthly mean antibiotic utilization rate (AUR) by 25% in participant NICUs.

**Result:**

Aggregate AUR was reduced by 15.3% (*p* < 0.001). There was a wide range in improvement among participant NICUs. There were no increases in EOS rates or nosocomial infection rates related to the intervention.

**Conclusion:**

Participation in this multicenter NICU antibiotic stewardship collaborative targeting EOS was associated with an aggregate reduction in antibiotic use. This study informs efforts aimed at sustaining improvements in NICU AURs.

## Introduction

Current rates of antibiotic use in neonatal intensive care units (NICUs) do not correlate with rates of proven infection, and overuse of antibiotics is common [1–5]. Antibiotic exposure in early life disrupts normal microbiome development and is associated with later development of diseases, including necrotizing enterocolitis, asthma, allergies, and obesity [6–9]. Antibiotic use promotes resistance [10], and is associated with increases in morbidity, mortality, length of hospital stay, and hospital costs [11–13]. Preterm neonates are particularly vulnerable to adverse effects of antibiotic exposure [14–20]. Management of suspected sepsis in NICUs evolved with the assumption of little or no adverse impact of antibiotics. New approaches to antibiotic use are needed to adjust to lower rates of early onset sepsis (EOS) related to maternal antibiotic prophylaxis. At the time of this study, there were no consensus early onset sepsis (EOS) guidelines that were focused on antibiotic stewardship. However, expert opinion suggested that traditional approaches to EOS antibiotic use likely resulted in overtreatment and new strategies were being encouraged [21].

National guidelines from the Centers for Disease Control and Prevention (CDC) [22] and The Joint Commission recommend healthcare system and hospital-level changes to improve antibiotic stewardship [23]. Specific methods of implementing these broad recommendations for individual NICUs requires further study. Limited evidence—primarily from single-site NICU antibiotic stewardship projects—suggests that antibiotic use rates can be safely reduced [24]. Minimizing unnecessary antibiotic use in NICUs on a wide scale can not only help minimize adverse effects, but also may improve value and optimize family centered care. The aim of this study was to reduce the antibiotic use rates in participant NICUs by targeting EOS antibiotic use.

## Methods

### Context

This study was conducted by the California Perinatal Quality Care Collaborative (CPQCC) from June 2016 to November 2017. The collaborative was expected to support up to 30 NICUs. Invitations to participate were sent to all 134 member California NICUs. Member NICUs have existing data agreements in place that were used during this study. NICUs were recruited and joined on a first-come, first-serve basis. Twenty-eight NICUs joined the collaborative. A fee was required to join the collaborative.

### Aims

The primary aim was to reduce the mean aggregate NICU antibiotic utilization rate (AUR) for all infants at participant NICUs by 25%. The secondary aim was to move 80% of the 28 sites into the baseline collaborative lowest quartile (AUR below 17.2%) by the end of the collaborative.

### Intervention

**Fig. 1:**
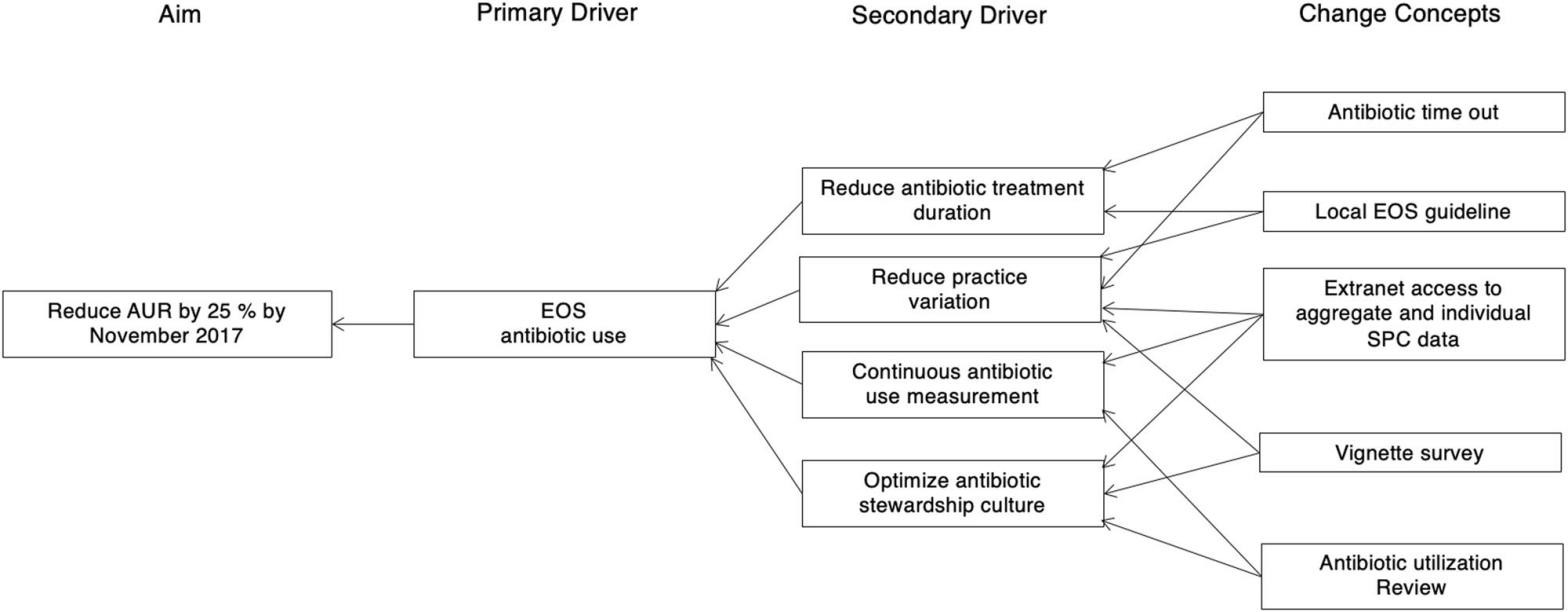
Driver Diagram. Driver diagram linking change concepts to a specific aim. AUR antibiotic utilization rate, SPC statistical process control, EOS early onset sepsis.

The intervention used methods described by the Institute of Healthcare Improvement Model for Achieving Breakthrough Improvement [25], and was consistent with prior CPQCC collaboratives [26–31]. A multidisciplinary advisory panel was established consisting of neonatologists, a neonatal hospitalist, infectious disease specialist, pharmacist, quality improvement specialist, clinical nurse specialist, and a parent representative. The panel identified key drivers of change (Fig. 1), developed a change package (Table 1) and measurement grid, led monthly webcasts, and facilitated e-mail listserv discussions. The panel chose to focus on EOS antibiotic usage because it was considered the most actionable area of antibiotic use in the NICU.

**Table 1:** Change package developed by expert panel.

1. Ensure appropriate discontinuation of antibiotics:
1a. Develop local early onset sepsis guidelines 1b. Establish routine “antibiotic time out” at 48 h (and no later than 72 h) after cultures are obtained
2. Establish real-time monitoring and measurement systems to ensure:
2a. Transparent availability of continuous antibiotic use data 2b. Data-informed feedback to individuals and groups of care providers
3. Promote a culture of optimal antibiotic use within the facility by:
3a. Convening a multidisciplinary group to perform periodic formal analysis of opportunities to improve antibiotic use

There were three face-to-face learning sessions during the 18-month collaborative, each attended by approximately 100 representatives from the 28 participant NICUs. A web-based survey using vignette research methods was conducted during the intervention period to identify and describe variation in individual antibiotic use for suspected EOS. Vignette results were reported in a separate manuscript [32]. Individual NICU vignette results were shared with sites that completed the vignettes. The results of the vignette assessment were used to guide discussion during the third learning session.

### Change package and driver diagram

Key drivers (Fig. 1) and change concepts (Table 1) were developed to reduce unwarranted EOS antibiotic use. Since there were no stewardship focused national EOS guidelines at the time of the study, the faculty panel and QIC leadership provided broad recommendations to participating NICUs without being prescriptive. This approach was used to encourage individual sites to focus on broad best practice themes without preventing individual site innovation hoping this approach would help identify optimal strategies unique to a given NICU’s local context. For example, the specific details of their time-out process were at their discretion and we did not require specific interventions outside the key drivers and change concepts.

### Timeline

Baseline/pre-intervention AUR data were collected for the dates June 1, 2015 to May 31, 2016 (the “baseline period”) and the intervention was implemented from June 1, 2016 to November 30, 2017 (the “intervention period”). The intervention period was further sub-divided into active intervention (June 1, 2016 to May 31, 2017) and sustainability (June 1, 2017 to November 30, 2017) subperiods.

### Study of the intervention

#### Measures

The primary outcome measure was monthly AUR, expressed as a proportion of days with antibiotic exposure. AUR was defined as the total number of patient days in which a patient in the NICU received one or more intravenous or intramuscular antibiotic doses divided by the total number of patient days during that month [2].

Process measures were the proportion of infants with an EOS evaluation who had documentation of completed antibiotic time out and monthly antibiotic usage review. A time-out was defined as any structured process that triggered prescribers to pause and consider discontinuation of antibiotics around 48 to 72 h after antibiotics were started. Balancing measures included annual EOS, nosocomial infection (NI), and mortality rates. These rates were retrospectively analyzed for correlation with reduction in AUR for each individual NICU to assess for unintended effects of the intervention. Participating sites entered outcome and process data into a password-protected collaborative-specific database. All sites were given access to the website to view their individual site data and aggregate data for the group.

Infection-related measures and mortality rates were obtained from California Children’s Services data that CPQCC member NICUs report annually. NICU characteristics were obtained from Vermont Oxford Network Annual Survey and data from Regional Perinatal Programs of California. These data were used to compare demographic and context characteristics of collaborative participant and non-participant NICUs to help determine the generalizability of results. Monthly AUR and antibiotic time-out data were collected from individual NICUs during the collaboration. Data collected from other entities and data collected from sites were linked within CPQCC’s database.

#### Data analysis

Participant and non-participant characteristics were analyzed using Fisher’s exact tests. The primary outcome measure of aggregate AUR was analyzed with a statistical process control Laney p’-chart to address overdispersion due to large numbers in each month subgroup. Individual NICU AURs were analyzed with p-charts. Montgomery chart rules were used for control chart analysis. Centerline and control limits were recalculated at the point where special cause variation was noted. Individual NICU chart centerline and control limits were recalculated for baseline, intervention, and sub-periods (active intervention, [June 2016–May 2017] and sustainability [June 2017–Nov 2017]). This analysis was performed to determine if improvements were temporary or sustained. Pre- and post-intervention AUR for aggregate and individual NICU AURs were compared to determine if there was an AUR reduction related to the intervention. A 3-month ramp-up period between baseline and intervention periods was excluded from the pre/post individual NICU comparison because most sites took several months to implement initial process changes. Individual p-charts were arranged in a small-multiples display to visualize and compare AUR variation among all NICUs.

Annual AUR change for each individual NICU was analyzed for correlation with change in annual EOS, NI, and mortality among all NICU admissions as the balancing measures. The Benjamini-Hochberg method with sequential Bonferroni correction was used to control for the false discovery rate related to multiple comparisons.

A mixed-methods quantitative and qualitative single-respondent context and process survey was conducted at the end of the intervention to determine characteristics of NICUs with >20% reduction between baseline and intervention median AUR. The 20% threshold was used to try to capture more NICUs since continuous AUR monitoring showed that many sites were not exceeding the 25% reduction mark several months into the collaborative. Median AUR was used to minimize impact of outliers. Qualitative analysis was performed to identify themes reported as most important for improvement in NICUs with >20% AUR reduction. ANOVA (continuous variables) and Fisher’s test (categorical variables) were used to compare differences among the three categories of improvement to identify characteristics of higher performers. NICU learning session report out presentations were reviewed as needed to explore individual NICU processes as needed.

This study was reviewed and approved by the Stanford University Institutional Review Board. Statistical analyses were performed using SAS 9.4 (Cary, North Carolina) and statistical process control charts were analyzed with QI macros 2017.09 (Denver, CO).

## Results

Twenty-eight (21%) of 134 eligible CPQCC member NICUs participated in this study. NICU characteristics are summarized in Table 2. Patient population characteristics and infection rate characteristics of participant and non-participant NICUs—comparing the means of both groups—are shown in Supplemental Table 3. The participant NICU group had a larger proportion of regional NICUs and level IV NICUs (Table 2), higher average daily census, greater mean NICU patient days, and higher mean total number of days with antibiotic exposures. The participant NICU group also had a higher mean number of live births, deaths, NICU admits, and surgical cases (Supplementary Table 3). Participant NICUs had a higher mean proportion of EOS cases per 1000 births (Supplementary Table 3).

**Table 2:** NICU characteristics of participant and non-participant NICUs.

Characteristics		Participants (*N* = 28)		Non-participants (*N* = 106)		
		*N*	%	*N*	%	*P* value
CCS Level	Non-CCS	1	3.6	15	14.2	**0.05**
	Intermediate	1	3.6	13	12.3	
	Community	17	60.7	64	60.4	
	Regional	9	32.1	14	13.2	
AAP Level	II	1	3.6	21	19.8	**0.029**
	III	19	67.9	71	67	
	IV	8	28.6	14	13.2	
Hospital Type	District	1	3.6	5	4.7	0.382
	For-Profit Private	3	10.7	12	11.3	
	Non-Profit Private	22	78.6	74	69.8	
	Public-City/County	0	0	11	10.4	
	University of California	2	7.1	4	3.8	
Academic Hospital	No	23	82.1	93	87.7	0.532
	Yes	5	17.9	13	12.3	

Significance test is based on Fisher’s exact test, comparing between study groups.

Hospital characteristics based on 2015 data.

At the beginning of the study five of the 28 NICUs allowed antibiotic treatment of term infants in the nursery. The other 23 NICUs treated term infants who required antibiotics in the NICU.

*CCS* California Children’s Services, *AAP* American Academy of Pediatrics.

**Fig. 2:**
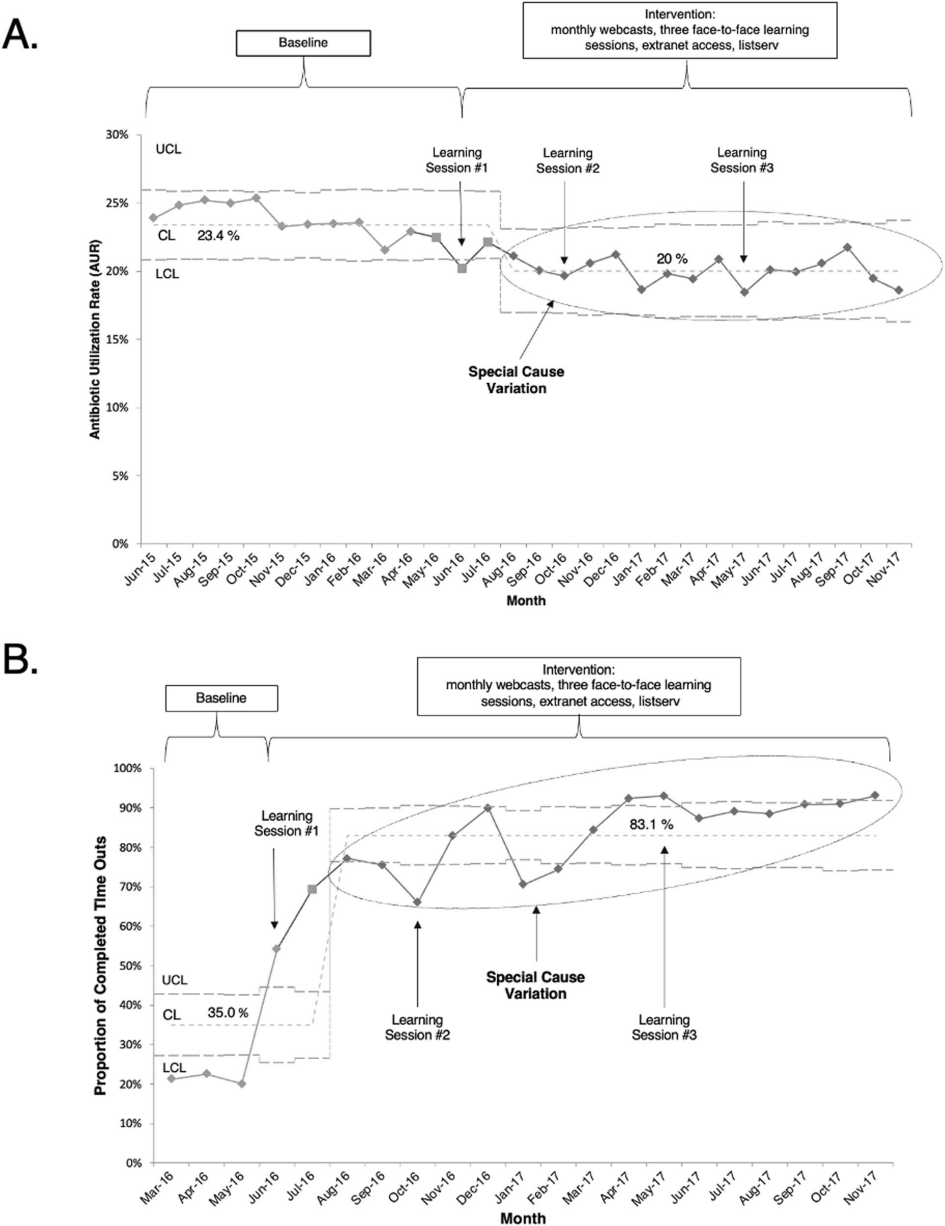
Statistical process control chart analysis of monthly aggregate AUR and monthly proportion of completed antibiotic time outs. **A** Statistical process control chart (Laney p’-chart) of monthly aggregate antibiotic utilization rates. The oval shape encircles months of AUR representing special cause variation with improved AUR. **B** Statistical process control chart (p-chart) of monthly proportion of completed antibiotic timeouts in infants evaluated for early onset sepsis. The oval shape encircles monthly time outs representing special cause variation with increased proportion of monthly time outs.UCL upper control limit, LCL lower control limit, CL centerline.

There was a 94% capture rate for monthly AURs among collaborative NICUs for the 18-month study period. Four NICUs did not submit AUR data during the sustainability period. Aggregate monthly AUR data from participant NICUs included 642,950 total patient days (270,246 baseline period days; 372,704 intervention period days). Special cause variation, with reduction in AUR, was first noted in the third month of the collaborative and was sustained for the remainder of the collaborative (Fig. 2A). Baseline mean AUR was 23.7% and was reduced to an intervention period mean AUR of 20.1%—a 15.3% reduction (*P* < 0.001) (Supplementary Table 4). This reduction reflects a mean AUR and percent reduction of 20.2% (15.1% reduction) and 20.1% (15.6% reduction) for the active and sustainability subperiods, respectively (Supplementary Table 4).

**Fig. 3:**
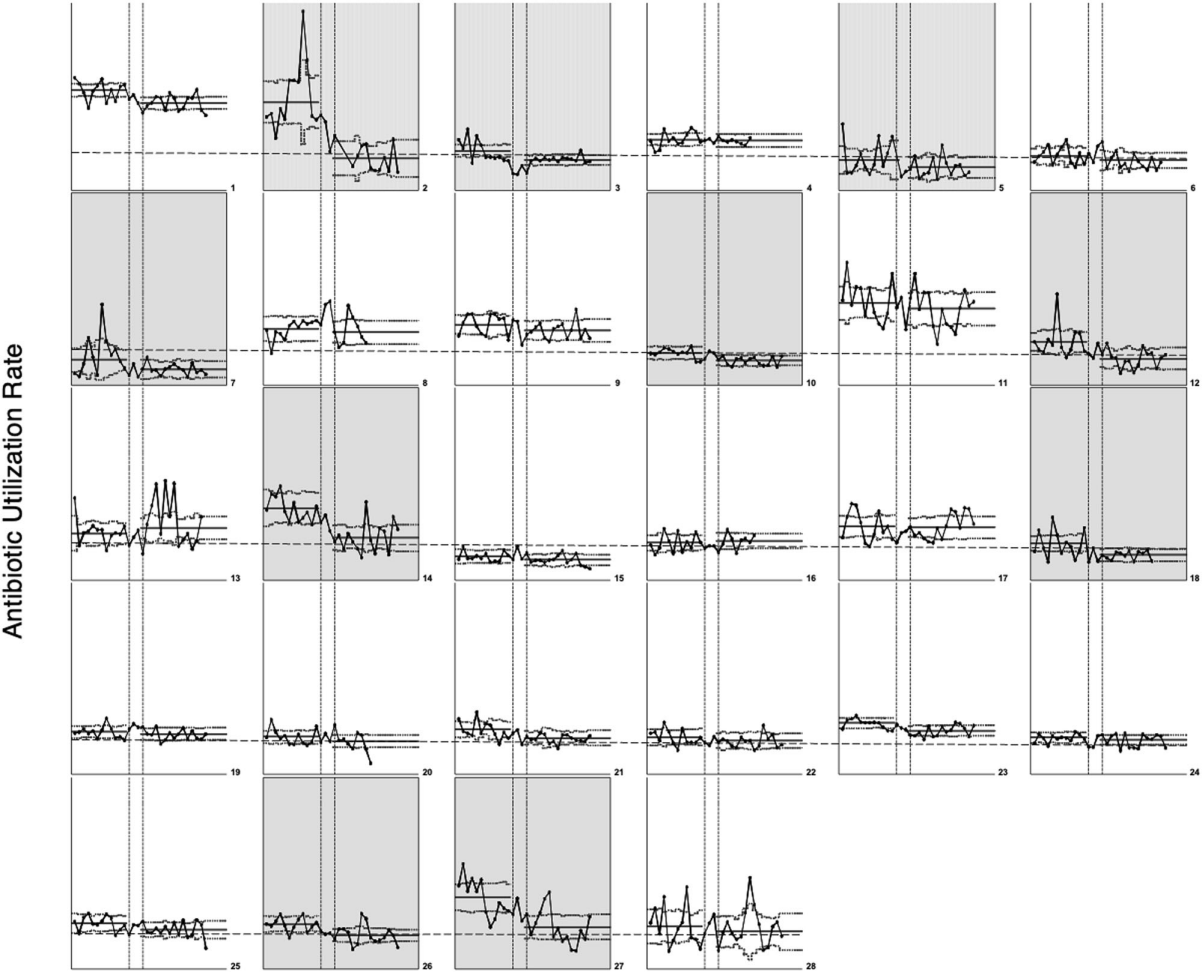
Small multiples display of individual NICU p-charts. The vertical axis shows the antibiotic utilization rate. The vertical scale is harmonized to allow comparison of NICUs in each row. The horizontal axis represents time and includes monthly baseline and intervention period AUR. The dashed line extending through each row marks the 17.2% AUR lowest baseline quartile. The shaded NICUs met the goal of >25% AUR reduction.

Ten (36%) of 28 participating NICUs reduced AUR by greater than 25% (Supplementary Table 5 and Fig. 3). Thirteen (46%) of the 28 sites moved into the baseline lowest AUR quartile of <17.2%. The range of change in AUR between baseline and intervention periods among individual NICUs varied from a 69.4% reduction to a 20.2% increase in AUR (Supplementary Table 5). There were five sites with >40% reduction in AUR and two sites with >50% (69.4% and 55.9%) reduction in AUR. Thirteen (46%) of the 28 sites reduced AUR by <25% and five (18%) of 28 sites did not show a statistically significant reduction in AUR between baseline and intervention periods (Supplementary Table 5).

Nine of 28 (32%) NICUs reduced AUR during the active intervention subperiod and maintained improvement through the sustainability subperiod until the end of the collaborative. Twelve of 28 (43%) NICUs significantly reduced AUR in the period of active intervention but did not sustain improvement through the sustainability period. Special cause variation with improvement in compliance with antibiotic timeouts was noted in August 2016, which was the third month of the study (Fig. 2B). One NICU showed an increase in annual mortality rate during the years compared. There were no increased rates of EOS or NI in any of the participant NICUs comparing annual infection rates before and during the collaborative. Figure 3 shows monthly AUR data for all 28 NICUs and highlights the NICUs with >25% reduction in a small-multiples display.

All 28 participating sites completed the context and process survey at the end of the study. Higher performing sites were less likely to have their EOS guideline embedded in the electronic medical record (*P* = 0.017) (Supplementary Table 6). Qualitative analysis of reported primary drivers of improvement in the 11 sites with >20% AUR reduction noted themes of multidisciplinary collaboration, leadership, and frequent antibiotic usage review as primary drivers of AUR reduction.

## Discussion

NICU antibiotic stewardship efforts have been underway for several years, but EOS-related antibiotic exposure still far exceeds rates of proven EOS [5]. Participation in this collaborative led to a collective 15.3% reduction in mean aggregate AUR without evidence of increased EOS or NI rates within individual participating NICUs. The AUR reduction was below the pre-specified aim. However, this study provides helpful antibiotic stewardship lessons for individual prescribers, NICU leaders, and multi-site QI efforts. This is the first multicenter NICU antibiotic stewardship collaborative that we are aware of reporting comprehensive monthly AURs enabling accurate assessment of improvement and sustainability. Approximately one-third of NICUs met the goal of reducing mean AUR by >25% and sustained the reduction until the end of the collaborative. Nearly half of the sites reduced AUR by >25% but did not sustain the AUR reduction. More than half of the NICUs did not meet the goal of >25% reduction. In 2021, 31 California NICUs joined a federally funded antibiotic stewardship quality improvement collaborative [33]. Most of them joined because they had opportunities to improve their antibiotic use rates. NICUs still have a sense of urgency to improve appropriate antibiotic use in NICUs. We explored the variable range of improvement in this study to provide helpful lessons for current and future NICU antibiotic stewardship efforts and studies.

Special cause variation, with reduced aggregate AUR, was noted during the third month of the collaborative. Each subsequent monthly AUR of the 18-month collaborative was below the pre-collaborative mean AUR. Although EOS antibiotic use does not reflect all antibiotic use in NICUs, the achievement of sustained improvement by one-third of participating NICUs in our collaborative suggests that EOS may be a reasonable area to begin stewardship efforts. However, a relatively small proportion of participant NICUs contributed to the collective improvement. Nearly half of the participating sites showed early improvement but were unable to sustain improvements during the relatively short time frame. NICU stewardship efforts should focus on addressing sustainability early and integrate processes into the standard work to increase the likelihood of maintaining improvement.

We visualized individual NICU monthly AUR charts, individual NICU range of performance, and contributions to the aggregate performance by displaying all NICU monthly AURs in a small multiples figure (Fig. 3). The figure highlights the challenge of wide monthly variability in identifying improvement. The display is an important supplement to accurately describe aggregate data as a product of individual NICU context and performance. We attempted to identify context and process factors associated with higher-performing NICUs. Leveraging the wide range in degree of improvement among the sites, we analyzed qualitative survey data to identify factors associated with higher-performing NICUs. Higher performing teams, with AUR reductions >20%, identified multidisciplinary collaboration, leadership, and frequent antibiotic usage review as primary drivers of improvement. However, our quantitative analysis of characteristics did not identify more specific context characteristics or processes associated with higher-performing NICUs. Participation in the collaborative may have only facilitated improvement in NICUs with local factors supporting readiness for improvement. Notably, one site (NICU # 13), showed an increase in AUR during the collaborative. This site reported their specific challenges during the last study webcast. When asked specifically about perceived contributors to the increased AUR, they reported continuation of antibiotics beyond seven days due to abnormal labs, lack of documentation of intent to discontinue antibiotics in initial admission assessment documentation, high patient acuity, and staffing challenges that limited daily antibiotic rounds and antibiotic review meetings.

Concern for missed infections, morbidity, and mortality related to delays or early discontinuation of antibiotics was a persistent concern noted throughout the study. Overall, this study did not detect increased annual EOS or NI rates related to the study period. Annual data showed that one site had an increase in all-cause mortality rate during the collaboration. However, as with all the other NICUs, this NICU did not have an increase in annual EOS or NI during the years of this study. Although more large-scale studies exploring the safety of antibiotic reductions are needed, these data support the safety of antibiotic stewardship in a large group of NICUs with AUR reductions over time without infection-related morbidity and mortality.

A cross-sectional study of antibiotic exposure in full-term and late preterm infants during the first week of life (among 11 countries in Europe, United States, and Australia, including the year after our study, through 2018) documented antibiotic exposure for EOS that is disproportionately high relative to rates of EOS [5]. They also noted high variability with a 9-fold difference in antibiotic exposure among sites. Given this recently published data, there is still a lot to learn about sustaining antibiotic use that is more consistent with rates of proven infection. Our study was conducted before the 2018 AAP guidelines for EOS in neonates were published [34, 35]. However, our study provides several practical lessons to address the continued overuse of antibiotics.

This study provides three important stewardship lessons for future large-scale NICU stewardship efforts and individual sites. Firstly, one NICU retrospectively stratified their AUR by isolating term infants and noted there was special cause variation with reduced AUR in this subgroup analysis that was not detected with AUR, including all gestational ages. Relying on AUR that includes all babies and all patient days may overlook important improvements that could signal initial success with a process change targeting a specific subgroup [36]. NICUs may benefit from stratifying antibiotic use measures by the specific patient characteristics and by time frame targeted by a given stewardship strategy to support sustained improvement. Secondly, as noted, our qualitative results highlighted frequent review of antibiotic use data as an important driver of improvement. Many sites could benefit from robust systems providing more frequent assessment of statistical process control analysis of antibiotic use. In 2021, none of 30 sites registered for the Optimizing Antibiotic Stewardship in California NICUs collaborative stewardship study were stratifying AUR into subgroups and very few were using automated capture of AUR with frequent statistical process control analysis. Current EMR infrastructure and statistical process control software make this process relatively easy and high value. A delay of 1–2 months to review time series AUR data can hinder stewardship efforts. Designating clinicians to perform this analysis at least monthly and marketing this data widely may be beneficial.

Lastly, individual practice variation in antibiotic decisions was a notable barrier raised by site QI teams. Individual practice variation is repeatedly noted as a barrier to providing optimal care, but is usually not formally addressed during QI efforts. Therefore, we used vignette research methods to identify and describe practice variation among prescribers at participating NICUs. There are wide ranging drivers and individual factors that determine whether and individual prescriber will start or stop antibiotics. The factorial vignette study results, described in a separate manuscript, objectively identified specific individual prescriber decisions as primary targets for further stewardship efforts [32]. The vignettes engaged prescribers who were not directly involved with QI teams, provided an objective description of variation, and allowed individuals to compare their practices among peers. Patient case simulations with feedback to providers have been shown to improve quality of care, compliance with evidence-based practices, and reduce costs [37, 38]. Vignettes may help reduce variation, improve stewardship practices, and sustain more appropriate AURs in NICUs if used at multiple time points during stewardship efforts [39]. Reflecting on our experience with this study, analyzing, interpreting, and sharing results from vignette assessments was an important supplemental collaborative quality improvement tool. Further research is required to determine effectiveness and ideal strategies for implementing vignette methods to support antibiotic stewardship in collaborative QI. However, vignette assessment should be considered for any QI project where practice variation is a notable barrier to improvement.

EMR-based interventions were not correlated with reduced AUR in this study, but EMR-based interventions have the potential to support stewardship [40, 41]. Significant improvements have occurred in EMRs in NICUs over the past 5 years with many sites transitioning to more sophisticated EMR platforms with a range of decision support and processes that could supplement stewardship efforts. The evidence supporting EMR interventions improving stewardship in hospitals is low quality, so the potential impact of NICU specific EMR processes targeting antibiotic stewardship is not known [42].

There was widespread adoption and compliance with antibiotic time outs over the course of the collaborative. As noted, this was one of the change package strategies recommended to sites, while leaving sites the autonomy to create their own specific time out process. For example, some sites adopted a time-out process as part of medical team sign-out. Some NICUs adopted a “third party” approach with a pharmacist directly approaching the medical team when the pharmacist performed their routine check of gentamicin levels around 48 h. In 2014, the CDC recommended a time out process within the core elements of antibiotic stewardship [22]. The 2018 AAP guidelines for the management of EOS, published after our study, recommend discontinuation of antibiotics at 36–48 h if there are negative blood cultures and no indication of site-specific infection [34, 35]. Our study supports that large scale implementation of antibiotic time out processes can be sustained over time. However, wide-scale successful implementation of EOS antibiotic time outs alone did not translate to the desired improvement for many sites. Thus, time outs may be one of several processes required to sustain more appropriate antibiotic use.

Effective strategies for externally facilitated NICU antibiotic stewardship programs and/or stewardship collaboratives have not been well studied. Dukovny et al. noted reduced antibiotic use in a 146 NICU multicenter antibiotic stewardship collaborative, but antibiotic use was only assessed by four single-day audits. They noted reduction in median AUR from a baseline of 16.7% to 12.1% [23]. Schulman et al.—with some AUR data overlapping with our study—retrospectively studied AURs in California NICUs and showed that NICUs participating in externally facilitated antibiotic stewardship projects had larger reductions in annual AUR compared to non-participants [4]. Our study and these studies suggest that externally facilitated stewardship programs may help optimize stewardship. However, identifying the optimal methods by which quality improvement collaboratives and individual NICUs improve and sustain more appropriate antibiotic use rates requires more rigorous quality improvement studies. These studies may require more detailed information on individual site context, individual prescriber decisions, timing of specific interventions, and longer sustainability follow-up periods.

This study has several limitations. The participating NICUs paid to join, which may introduce selection bias for NICUs with relatively more resources and high levels of motivation for change. Participating NICUs had different characteristics relative to non-participant NICUs in California, which could limit the generalizability of our results. We did not have an adequate control group with monthly AUR data to compare performance among other NICUs. There was a downward trend in aggregate AUR leading up to the start of the collaborative, which limits our ability to ascribe the improvement to the intervention. Although we were unable to collect the pre-planned balancing measures during the study, our retrospective linkage of individual NICU annual infection data provides a more robust balancing measure assessment than our pre-specified and commonly used measures of “restarting antibiotics” and “readmissions” for antibiotic treatment.

## Conclusions

Participation in this statewide multicenter NICU antibiotic stewardship collaborative led to a reduction in aggregate AUR without apparent increased infections or infection-related mortality. Some participating NICUs did not meet the AUR reduction goal and some NICUs that improved did not sustain AUR reductions. This study provides specific, actionable strategies for NICU antibiotic stewardship. Future studies should explore optimal strategies to sustain reduced AURs in NICUs.

### Supplementary information

Supplemental Material

Supplemental Table 5
