# Supplementary material for: 28 NICUs participating in a quality improvement collaborative targeting early-onset sepsis antibiotic use
Source: J Perinatol. 2024 Feb 20;44(7):1061–8. doi: 10.1038/s41372-024-01885-8 (PMC11226396; doi:10.1038/s41372-024-01885-8)
Supplement: Supplementary file 1 — Supplemental Material [file 41372_2024_1885_MOESM1_ESM.docx]

| Supplemental Table 1: Patient characteristics and infection rate characteristics of participant and non-participant NICUs | Non-participants (N=106) | | | | |  | Participants (N=28) | | | | |  |
| --- | --- | --- | --- | --- | --- | --- | --- | --- | --- | --- | --- | --- |
| Variable | N | Mean | STD | Median | IQR |  | N | Mean | STD | Median | IQR | P-value^a^ |
| Average Daily Census | 106 | 13.2 | 11.4 | 10.2 | 6.4-16.1 |  | 28 | 26.8 | 18.9 | 22.3 | 12.4-33.2 | **<.001** |
| Total NICU Patient Days | 106 | 4812.1 | 4146.8 | 3735.5 | 2347-5887 |  | 28 | 9784.6 | 6894.7 | 8139.5 | 4512.5-12115 | **<.001** |
| % NICU Days w/ Antibiotic Exposure | 105 | 25.0 | 13.0 | 22.2 | 14.4-34.5 |  | 28 | 22.6 | 8.0 | 21.2 | 16.6-26.3 | 0.357 |
| Total Live Births | 106 | 2579.1 | 1351.3 | 2431.5 | 1796-3300 |  | 28 | 3373.9 | 2036.0 | 3365.0 | 2054.5-4046.5 | **0.015** |
| Total Deaths | 106 | 4.7 | 8.4 | 2.0 | 0-4 |  | 28 | 11.7 | 14.8 | 6.5 | 2-14.5 | **0.001** |
| Total NICU Admits | 106 | 367.0 | 217.9 | 323.5 | 221-429 |  | 28 | 645.8 | 368.2 | 532.0 | 414-858.5 | **<.001** |
| Early Sepsis per 1,000 live births | 103 | 0.5 | 0.6 | 0.4 | 0-0.7 |  | 26 | 0.9 | 0.9 | 0.6 | 0.2-1.4 | **0.018** |
| % Nosocomial Infection | 105 | 3.7 | 3.8 | 2.9 | 0-5.6 |  | 28 | 4.2 | 3.8 | 3.9 | 2.5-5 | 0.547 |
| % Coagulase negative staph | 105 | 1.8 | 2.6 | 0.4 | 0-2.6 |  | 28 | 1.9 | 2.6 | 1.3 | 0.3-2.4 | 0.767 |
| % Necrotizing enterocolitis (SB) | 106 | 2.4 | 3.6 | 0.0 | 0-4.8 |  | 28 | 3.7 | 5.8 | 2.2 | 0.5-4.7 | 0.153 |
| Total number of surgeries | 106 | 30.8 | 90.1 | 1.0 | 0-12 |  | 28 | 82.1 | 140.1 | 13.5 | 0-84 | **0.020** |
|  |  |  |  |  |  |  |  |  |  |  |  |  |
|  |  |  |  |  |  |  |  |  |  |  |  |  |
| NICU, Neonatal Intensive Care Unit; SB, small babies (<31/67 weeks, <1500 grams); N, number of NICUs; IQR, interquartile range  ^a^ P-values comparing participant with non-participant group in the same time period, based on T-test (continuous variables). | | | | | | | |  |  |  |  |  |

Supplemental Table 2: Aggregate antibiotic utilization rates (AURs) during baseline, intervention, and sustainability periods

|  |  |  | |  |  | |  | |  |  | |  | |  | |  |  |  |  |  |  |  |  |
| --- | --- | --- | --- | --- | --- | --- | --- | --- | --- | --- | --- | --- | --- | --- | --- | --- | --- | --- | --- | --- | --- | --- | --- |
|  | | |  | | |  | |  | | |  | |  | |  | |  |  |  |  |  |  |  |
| Study period | | |  | | | Number of NICUs | | Total patient days | | | Total exposure days | | Total  AUR^a^ | | Mean AUR^b^ | | Median AUR^b^ | Min AUR | Max AUR | %Reduction from baseline mean AUR | P- value |  |  |
| Baseline | | | June 2015-May 2016 | | | 28 | | 270,246 | | | 64,187 | | 23.8% | | 23.7% | | 23.5% | 21.6% | 25.3% | -- | -- |  |  |
| Intervention | | | June 2016-May 2017 | | | 28 | | 264,221 | | | 53,369 | | 20.2% | | 20.2% | | 20.1% | 18.4% | 22.1% | 15.1% | **<.001** |  |  |
| Sustainability | | | June 2017-Nov 2017 | | | 24 | | 108,483 | | | 21,772 | | 20.1% | | 20.0% | | 20.0% | 18.6% | 21.7% | 15.6% | **<.001** |  |  |
|  | | |  | | |  | |  | | |  | |  | |  | |  |  |  |  |  |  |  |
| Intervention + Sustainability | | | Jun 2016-Nov 2017 | | | 28 | | 372,704 | | | 75,141 | | 20.2% | | 20.1% | | 20.1% | 18.4% | 22.1% | 15.3% | **<.001** |  |  |
|  | | |  | | |  | |  | | |  | |  | |  | |  |  |  |  |  |  |  |
|  | | |  | | |  | |  | | |  | |  | |  | |  |  |  |  |  |  |  |
|  | | |  | | |  | |  | | |  | |  | |  | |  |  |  |  |  |  |  |
|  | | |  | | |  | |  | | |  | |  | |  | |  |  |  |  |  |  |  |
|  | | |  | | |  | |  | | |  | |  | |  | |  |  |  |  |  |  |  |
|  | | | | | | | |  | | |  | |  | |  | |  |  |  |  |  |  |  |
|  | | |  | | |  | |  | | |  | |  | |  | |  |  |  |  |  |  |  |
|  | | | | | | | |  | | |  | |  | |  | |  |  |  |  |  |  |  |

^a^ Total AUR reflects AUR for all months combined within each study period

^b^ Mean and median AUR were obtained from monthly AUR for each study period

AUR, antibiotic utilization rate; NICU, neonatal intensive care unit; Min, minimum; Max, maximum

Supplemental Table 3: Change in individual NICU antibiotic utilization rates

|  | |  |  |  |  |
| --- | --- | --- | --- | --- | --- |
|  |  |  |  |  |  |
| NICU | Baseline^a^ | Intervention^b^ | Difference^c^ | % Change | P-value |
| 1 | 50.9% | 44.0% | -6.9% | -13.6% | **<.001** |
| 2 | 49.3% | 15.1% | -34.2% | -69.4%* | **<.001** |
| 3 | 19.0% | 14.1% | -4.9% | -26.0%* | **<.001** |
| 4 | 24.7% | 23.8% | -0.9% | -3.5% | 0.309 |
| 5 | 15.6% | 9.8% | -5.8% | -36.9%* | **<.001** |
| 6 | 16.0% | 12.8% | -3.2% | -19.8% | **<.001** |
| 7 | 13.8% | 6.1% | -7.7% | -55.9%* | **<.001** |
| 8 | 27.3% | 26.1% | -1.2% | -4.3% | 0.109 |
| 9 | 30.0% | 26.4% | -3.6% | -11.9% | **<.001** |
| 10 | 14.8% | 10.5% | -4.3% | -29.0%* | **<.001** |
| 11 | 43.0% | 38.0% | -5.0% | -11.6% | **0.026** |
| 12 | 21.4% | 11.8% | -9.6% | -44.9%* | **<.001** |
| 13 | 23.6% | 28.3% | 4.8% | 20.2% | **0.002** |
| 14 | 36.3% | 20.6% | -15.7% | -43.2%* | **<.001** |
| 15 | 9.8% | 8.1% | -1.8% | -18.1% | **<.001** |
| 16 | 17.2% | 18.0% | 0.8% | 4.8% | 0.131 |
| 17 | 25.9% | 25.8% | -0.1% | -0.3% | 0.555 |
| 18 | 17.2% | 10.9% | -6.3% | -36.7%* | **<.001** |
| 19 | 20.0% | 18.8% | -1.2% | -5.8% | **0.016** |
| 20 | 17.6% | 14.9% | -2.7% | -15.3% | **<.001** |
| 21 | 21.2% | 16.6% | -4.6% | -21.7% | **<.001** |
| 22 | 17.4% | 15.1% | -2.3% | -13.0% | **<.001** |
| 23 | 24.6% | 20.7% | -3.9% | -16.0% | **<.001** |
| 24 | 16.9% | 15.7% | -1.2% | -7.2% | **0.002** |
| 25 | 22.1% | 18.4% | -3.6% | -16.5% | **<.001** |
| 26 | 22.0% | 16.1% | -5.9% | -26.7%* | **<.001** |
| 27 | 36.7% | 20.5% | -16.2% | -44.1%* | **<.001** |
| 28 | 20.3% | 19.6% | -0.6% | -3.0% | **0.019** |
|  |  |  |  |  |  |
|  | | |  |  |  |

^a^ Baseline/pre-intervention: June 1, 2015 to May 31, 2016

^b^ Intervention: June 1,2016 — November 30, 2017

^c^ Three month ramp up period excluded from baseline to intervention comparison analysis (June 1, 2016 – August 31,2016)

^*^ Greater than 25% reduction

| NICU characteristics | >20%  AUR  reduction  (N = 11) | 10-20%  AUR  reduction  (N = 5) | <10%  AUR  reduction  (N = 12) | P-value |
| --- | --- | --- | --- | --- |
| NICU Beds | 27 (12) | 57 (26) | 33 (19) | **0.017** |
| AAP Level II | 1 (9.1%) | 1 (20%) | 0 (0%) | 0.264 |
| III | 7 (63.6%) | 1 (20%) | 8 (66.7%) | -- |
| IV | 3 (27.3%) | 3 (60%) | 4 (33.3%) | -- |
| Total Live Births; mean (SD) | 3,090 (1878) | 3,893 (3421) | 3,148 (1504) | 0.752 |
| Total NICU admits; mean (SD) | 494 (307) | 902 (435) | 586 (231) | 0.060 |
| Average Daily Census; mean (SD) | 18.4 (9.6) | 40.6 (3.02) | 25.7 (18.3) | 0.110 |
| Primary reason to join the collaborative:  Always participate CPQCC collaborative  High antibiotic use at site | 7 (64%)  1 (9%) | 3 (60%)  3 (60%) | 8 (67%)  5 (42%) | 1.000  0.078 |
| Number of members on your Antibiotic Stewardship Team: |  |  |  |  |
| Neonatologists | 3.5 (4.0) | 2.6 (1.1) | 3.0 (2.5) | 0.831 |
| Registered Nurse | 2.4 (1.4) | 1.6 (1.3) | 1.3 (0.6) | 0.067 |
| Total | 8.0 (5.0) | 6.0 (2.9) | 7.6 (4.5) | 0.711 |
| Number of providers at your site:  Neonatologists  Neonatal Nurse Practitioners  Total | 8.3 (6.6)  1.3 (2.6)  19 (24) | 11.6 (6.5)  4.4 (5.4)  44 (71) | 7.6 (4.5)  4.1 (6.6)  34 (43) | 0.409  0.360  0.510 |
| What is in included in your current early onset sepsis guideline? (check all that apply):  Written/documented Sepsis Guideline  Guideline is embedded in EMR | 9 (82%)  0 (0%) | 2 (40%)  3 (60%) | 9 (75%)  3 (25%) | 0.262  **0.017** |
| Components included in audits of antibiotic use (check all that apply):  Intermittent chart review  Prospective/Retrospective chart review for all antibiotic use >48 hours  Physician driven | 5 (45%)  6 (55%)  5 (45%) | 2 (40%)  3 (60%)  2 (40%) | 5 (42%)  5 (42%)  3 (25%) | 1.000  0.787  0.589 |
| AUR feedback to providers (check all that apply):  Formal face to face  Formal email based  From Antibiotic Stewardship Team  From NICU leadership | 4 (36%)  4 (36%)  6 (55%)  5 (46%) | 3 (60%)  1 (20%)  2 (40%)  2 (40%) | 2 (17%)  3 (25%)  5 (42%)  3 (25%) | 0.183  0.868  0.886  0.589 |

Supplemental Table 4: Characteristics of NICUs by categorized by degree median antibiotic utilization rate reduction

ANOVA and Fisher’s test were used to compare differences among the three categories of improvement to identify characteristics of higher performers

NICU, Neonatal Intensive Care Unit; SD, Standard Deviation; AAP American Academy of Pediatrics; CPQCC, California Perinatal Quality Care Collaborative; RN, registered nurse; EMR, electronic medical record
