# Supplementary material for: 28 NICUs participating in a quality improvement collaborative targeting early-onset sepsis antibiotic use
Source: J Perinatol. 2024 Feb 20;44(7):1061–8. doi: 10.1038/s41372-024-01885-8 (PMC11226396; doi:10.1038/s41372-024-01885-8)
Supplement: Supplementary file 2 — Supplemental Table 5 [file 41372_2024_1885_MOESM2_ESM.docx]

Supplemental Table 5: Change in individual NICU antibiotic utilization rates

|  | |  |  |  |  |
| --- | --- | --- | --- | --- | --- |
|  |  |  |  |  |  |
| NICU | Baseline^a^ | Intervention^b^ | Difference^c^ | % Change | P-value |
| 1 | 50.9% | 44.0% | -6.9% | -13.6% | **<.001** |
| 2 | 49.3% | 15.1% | -34.2% | -69.4%* | **<.001** |
| 3 | 19.0% | 14.1% | -4.9% | -26.0%* | **<.001** |
| 4 | 24.7% | 23.8% | -0.9% | -3.5% | 0.309 |
| 5 | 15.6% | 9.8% | -5.8% | -36.9%* | **<.001** |
| 6 | 16.0% | 12.8% | -3.2% | -19.8% | **<.001** |
| 7 | 13.8% | 6.1% | -7.7% | -55.9%* | **<.001** |
| 8 | 27.3% | 26.1% | -1.2% | -4.3% | 0.109 |
| 9 | 30.0% | 26.4% | -3.6% | -11.9% | **<.001** |
| 10 | 14.8% | 10.5% | -4.3% | -29.0%* | **<.001** |
| 11 | 43.0% | 38.0% | -5.0% | -11.6% | **0.026** |
| 12 | 21.4% | 11.8% | -9.6% | -44.9%* | **<.001** |
| 13 | 23.6% | 28.3% | 4.8% | 20.2% | **0.002** |
| 14 | 36.3% | 20.6% | -15.7% | -43.2%* | **<.001** |
| 15 | 9.8% | 8.1% | -1.8% | -18.1% | **<.001** |
| 16 | 17.2% | 18.0% | 0.8% | 4.8% | 0.131 |
| 17 | 25.9% | 25.8% | -0.1% | -0.3% | 0.555 |
| 18 | 17.2% | 10.9% | -6.3% | -36.7%* | **<.001** |
| 19 | 20.0% | 18.8% | -1.2% | -5.8% | **0.016** |
| 20 | 17.6% | 14.9% | -2.7% | -15.3% | **<.001** |
| 21 | 21.2% | 16.6% | -4.6% | -21.7% | **<.001** |
| 22 | 17.4% | 15.1% | -2.3% | -13.0% | **<.001** |
| 23 | 24.6% | 20.7% | -3.9% | -16.0% | **<.001** |
| 24 | 16.9% | 15.7% | -1.2% | -7.2% | **0.002** |
| 25 | 22.1% | 18.4% | -3.6% | -16.5% | **<.001** |
| 26 | 22.0% | 16.1% | -5.9% | -26.7%* | **<.001** |
| 27 | 36.7% | 20.5% | -16.2% | -44.1%* | **<.001** |
| 28 | 20.3% | 19.6% | -0.6% | -3.0% | **0.019** |
|  |  |  |  |  |  |
|  | | |  |  |  |

^a^ Baseline/pre-intervention: June 1, 2015 to May 31, 2016

^b^ Intervention: June 1,2016 — November 30, 2017

^c^ Three month ramp up period excluded from baseline to intervention comparison analysis (June 1, 2016 – August 31,2016)

^*^ Greater than 25% reduction

| NICU characteristics | >20%  AUR  reduction  (N = 11) | 10-20%  AUR  reduction  (N = 5) | <10%  AUR  reduction  (N = 12) | P-value |
| --- | --- | --- | --- | --- |
| NICU Beds | 27 (12) | 57 (26) | 33 (19) | **0.017** |
| AAP Level II | 1 (9.1%) | 1 (20%) | 0 (0%) | 0.264 |
| III | 7 (63.6%) | 1 (20%) | 8 (66.7%) | -- |
| IV | 3 (27.3%) | 3 (60%) | 4 (33.3%) | -- |
| Total Live Births; mean (SD) | 3,090 (1878) | 3,893 (3421) | 3,148 (1504) | 0.752 |
| Total NICU admits; mean (SD) | 494 (307) | 902 (435) | 586 (231) | 0.060 |
| Average Daily Census; mean (SD) | 18.4 (9.6) | 40.6 (3.02) | 25.7 (18.3) | 0.110 |
| Primary reason to join the collaborative:  Always participate CPQCC collaborative  High antibiotic use at site | 7 (64%)  1 (9%) | 3 (60%)  3 (60%) | 8 (67%)  5 (42%) | 1.000  0.078 |
| Number of members on your Antibiotic Stewardship Team: |  |  |  |  |
| Neonatologists | 3.5 (4.0) | 2.6 (1.1) | 3.0 (2.5) | 0.831 |
| Registered Nurse | 2.4 (1.4) | 1.6 (1.3) | 1.3 (0.6) | 0.067 |
| Total | 8.0 (5.0) | 6.0 (2.9) | 7.6 (4.5) | 0.711 |
| Number of providers at your site:  Neonatologists  Neonatal Nurse Practitioners  Total | 8.3 (6.6)  1.3 (2.6)  19 (24) | 11.6 (6.5)  4.4 (5.4)  44 (71) | 7.6 (4.5)  4.1 (6.6)  34 (43) | 0.409  0.360  0.510 |
| What is in included in your current early onset sepsis guideline? (check all that apply):  Written/documented Sepsis Guideline  Guideline is embedded in EMR | 9 (82%)  0 (0%) | 2 (40%)  3 (60%) | 9 (75%)  3 (25%) | 0.262  **0.017** |
| Components included in audits of antibiotic use (check all that apply):  Intermittent chart review  Prospective/Retrospective chart review for all antibiotic use >48 hours  Physician driven | 5 (45%)  6 (55%)  5 (45%) | 2 (40%)  3 (60%)  2 (40%) | 5 (42%)  5 (42%)  3 (25%) | 1.000  0.787  0.589 |
| AUR feedback to providers (check all that apply):  Formal face to face  Formal email based  From Antibiotic Stewardship Team  From NICU leadership | 4 (36%)  4 (36%)  6 (55%)  5 (46%) | 3 (60%)  1 (20%)  2 (40%)  2 (40%) | 2 (17%)  3 (25%)  5 (42%)  3 (25%) | 0.183  0.868  0.886  0.589 |

Supplemental Table 6: Characteristics of NICUs by categorized by degree median antibiotic utilization rate reduction

ANOVA and T-tests were used to compare differences among the three categories of improvement

NICU, Neonatal Intensive Care Unit; SD, Standard Deviation; AAP American Academy of Pediatrics; CPQCC, California Perinatal Quality Care Collaborative; RN, registered nurse; EMR, electronic medical record
